# Low-temperature synthesis of multilayer graphene/amorphous carbon hybrid films and their potential application in solar cells

**DOI:** 10.1186/1556-276X-7-453

**Published:** 2012-08-11

**Authors:** Tongxiang Cui, Ruitao Lv, Zheng-Hong Huang, Hongwei Zhu, Yi Jia, Shuxiao Chen, Kunlin Wang, Dehai Wu, Feiyu Kang

**Affiliations:** 1 Laboratory of Advanced Materials, Department of Materials Science and Engineering, Tsinghua University, Beijing, 100084, China; 2 Department of Physics, The Pennsylvania State University, University Park, PA, 16802, USA; 3 Key Laboratory for Advanced Manufacturing by Materials Processing Technology of the Ministry of Education, Department of Mechanical Engineering, Tsinghua University, Beijing, 100084, China; 4 Center for Nano and Micro Mechanics, Tsinghua University, Beijing, 100084, China; 5 Graduate School at Shenzhen, Tsinghua University, Shenzhen, Guangdong Province, 518055, China

**Keywords:** Graphene, Amorphous carbon, Temperature effect, Nickel foil, Solar cell, HNO_3_ treatment

## Abstract

The effect of reaction temperature on the synthesis of graphitic thin film on nickel substrate was investigated in the range of 400°C to 1,000°C. Amorphous carbon (a-C) film was obtained at 400°C on nickel foils by chemical vapor deposition; hybrid films of multilayer graphene (MLG) and a-C were synthesized at a temperature of 600°C, while MLG was obtained at temperatures in excess of 800°C. Schottky-junction solar cell devices prepared using films produced at 400°C, 600°C, 800°C, and 1,000°C coupled with n-type Si demonstrate power conversion efficiencies of 0.003%, 0.256%, 0.391%, and 0.586%, respectively. A HNO_3_ treatment has further improved the efficiencies of the corresponding devices to 0.004%, 1.080%, 0.800%, and 0.820%, respectively. These films are promising materials for application in low-cost and simple carbon-based solar cells.

## Background

Graphene has attracted widespread attention due to its unique band structure and fascinating electronic, optical, chemical, and mechanical properties [1-4]. Hybrid structures of graphene with other carbon materials such as carbon nanotubes (CNTs) [5,6] and amorphous carbon (a-C) [6] could combine advantages of the constituent structures and find applications in many areas. For example, composites of a-C and graphene sheets exhibit attractive catalytic performance for the hydrolysis of cellohexaose [7]. Multilayer graphene (MLG) oxide and a-C hybrid films were synthesized by incorporating MLG oxide into a-C matrix [8]. The hybrid films show good electrical, mechanical, and tribological properties, with a sheet resistivity of approximately 100 Ω cm, Young’s modulus of 171 GPa, and elastic recovery of 81.4% [8]. In addition, heterojunction solar cells based on carbon materials such as a-C films and graphene have attracted much attention [9]. Their interesting optical properties, chemical inertness, and low cost make a-C films as potential candidate materials for solar cells [10,11]. Indeed, carbon films were the earliest carbon materials partially replacing silicon (Si) in Si-based solar cells [9], but their poor electrical conductivity hinders their practical application [12]. Excellent conductivity, good transparency, and high hole transport mobility make graphene as a promising candidate in photovoltaic devices [9,13,14]. Graphene/n-Si Schottky-junction solar cells have been assembled and demonstrated power conversion efficiencies up to 1.5% [13], but the fabrication process of the device is relatively complex because graphene easily cracks [15]. Some of the above-mentioned shortcomings can be overcome by a graphene/a-C hybrid structure. Unfortunately, the reported method to prepare graphene/a-C hybrid structure involves several rigorous processing steps, including the fabrication of graphene oxide using expandable graphite, dissolving the graphene oxide into methanol, and electrolysis deposition of the methanol solution [8]. Therefore, *in situ* graphene/a-C hybrid structure fabrication is highly desirable.

The synthesis of carbon nanomaterials (carbon nanotubes, graphene, carbon films, etc.) at a relatively low temperature is crucial for their practical applications [16,17]. Firstly, a low temperature could simplify the growth process [18], and it is more convenient, cost-effective, and environment-friendly [16]. Secondly, a low-temperature process is important for their electronic device applications [19]. For example, complementary metal-oxide semiconductor (CMOS) technology is widely used in transistors. In CMOS technology, an oxide layer serves as an insulator between the transistor gate and the channel [20]. In this sense, synthesis of carbon nanomaterials directly on certain substrates (e.g., oxide [20], nickel [13,21]) is crucial. However, many substrates are vulnerable to heating [22]; thus, low-temperature synthesis of carbon nanomaterials would be attractive.

In this work, hybrid films of MLG and a-C were prepared at a relatively low temperature of 600°C. When the temperature exceeded 800°C, MLG is obtained. Schottky-junction solar cells based on n-type Si and 400°C, 600°C, 800°C, and 1,000°C samples demonstrate efficiencies of 0.003%, 0.256%, 0.391%, and 0.586%, respectively. After a HNO_3_ treatment, the efficiencies of the corresponding solar cells have further increased to 0.004%, 1.080%, 0.800%, and 0.820%, respectively. Our work has opened up a new avenue for the production of low-cost carbon-based solar cells in the future.

## Methods

### Synthesis of MLG

The experimental setup is similar to that for N-doped carbon films (N-CFMs) described in our previous work [23], except that H_2_ is used in this work. In addition, the substrate is also different from that of N-CFMs. Nickel (Ni) foil instead of copper foil is used as substrate for graphene growth. The Ni foil is mounted in the center of the quartz tube reactor and gradually heated up to the pre-determined temperature of 400°C, 600°C, 800°C, or 1,000°C in 100 min under the protection of an Ar flow of 300 ml/min. When the temperature reaches the set value, the Ni foil is further annealed for 30 min to homogenize the crystal grains and to remove the oxidation layers under a reducing atmosphere of Ar (2,000 ml/min) mixed with H_2_ (100 ml/min). Then, acetonitrile (CH_3_CN) is introduced into the reactor at a feed rate of 20 μl/min for 2 min under the reducing atmosphere of Ar (2,000 ml/min) and H_2_ (100 ml/min). Afterwards, the Ni foil is moved to the low-temperature region of the quartz reactor to achieve a fast cooling rate. To obtain freestanding films, the as-grown samples are treated in a mixed solution of 0.5 M FeCl_3_ and 0.5 M HCl. After being rinsed in distilled water for several times, freestanding films could be collected for further characterizations and device fabrication.

### Characterization of samples

The morphologies of as-synthesized films were characterized using a transmission electron microscope (TEM, JEOL-2010, Akishima-shi, Japan). Raman spectra were obtained on a microscopic confocal Raman spectrometer (Renishaw RM 2000, Wotton-under-Edge, UK) with a 514.5-nm laser line. Optical transmission spectra were taken using a UV-2450 UV/Vis optical spectrometer (Shimadzu Corporation, Kyoto, Japan). Sheet resistances (*R*_s_) of the samples were measured using a four-probe resistivity test system.

### Solar cell device assembly

Heterojunction solar cells were assembled by covering the films synthesized at 400°C, 600°C, 800°C, and 1,000°C onto an n-type Si wafer with a square window of 3 mm × 3 mm surrounded by insulating silicon dioxide, and the detailed procedure of the cell assembling can be found in our previous report [23]. The assembled solar cells were evaluated with a solar simulator (at AM 1.5, Newport, Irvine, CA, USA) and a Keithley 2400 SourceMeter (Cleveland, OH, USA).

### HNO_3_ treatment

The HNO_3_ treatment was carried out by exposing the as-synthesized films to HNO_3_ fumes. The assembled solar cell was placed above a vial containing fuming HNO_3_ (65 wt.%) for 60 s.

## Results and discussion

**Figure 1:**
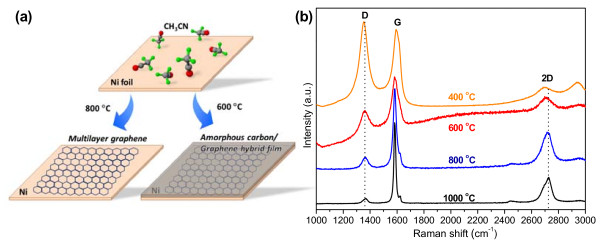
**Illustration and Raman spectra of as-grown films at different temperatures.** (**a**) Illustration of films produced at different reaction temperatures. (**b**) Raman spectra of as-grown films obtained at different temperatures.

An illustration of products synthesized at different temperatures and their typical Raman spectra are shown in Figure 1a,b, respectively. The spectra of all the products (Figure 1b) consist of three peaks at 1,350, 1,590, and 2,700 cm^−1^, which correspond to the D-band, G-band, and 2D-band, respectively. The D-band is activated usually by disorder and grain boundaries. It can be clearly seen from Figure 1b that the D-band becomes weaker as temperature increases, which indicates a decrease of defects with an increasing temperature. The G-band depends on the in-plane stretching motion and occurs for all *sp*^2^ carbon atom sites [24]. Obvious G-band could be seen in products at all temperatures, which indicates the formation of *sp*^*2*^ carbon bondings. Sharp and symmetrical 2D-band is a typical feature of monolayer graphene [25,26]. The full width at half maximum (FWHM) of a monolayer of graphene is approximately 30 cm^−1^, and the *I*_2D_/*I*_G_ ratio is approximately 3 [25]. There is a significant difference in the line shape of the 2D-band between samples obtained at low temperature (400°C and 600°C) and high temperature (800°C and 1,000°C). The 800°C and 1,000°C samples have a FWHM of 86 cm^−1^ and an *I*_2D_/*I*_G_ ratio of approximately 0.45, showing the Raman feature of MLG [24]. The 400°C and 600°C samples do not show obvious 2D-band. Furthermore, an obvious downshift of the 2D-band of the low-temperature samples (approximately 2,708 cm^−1^) could be seen, which should be compared with approximately 2,722 cm^−1^ for high-temperature ones. The difference of 2D-band between these samples is attributed to their difference in structures, which is confirmed by subsequent TEM measurements. 

**Figure 2:**
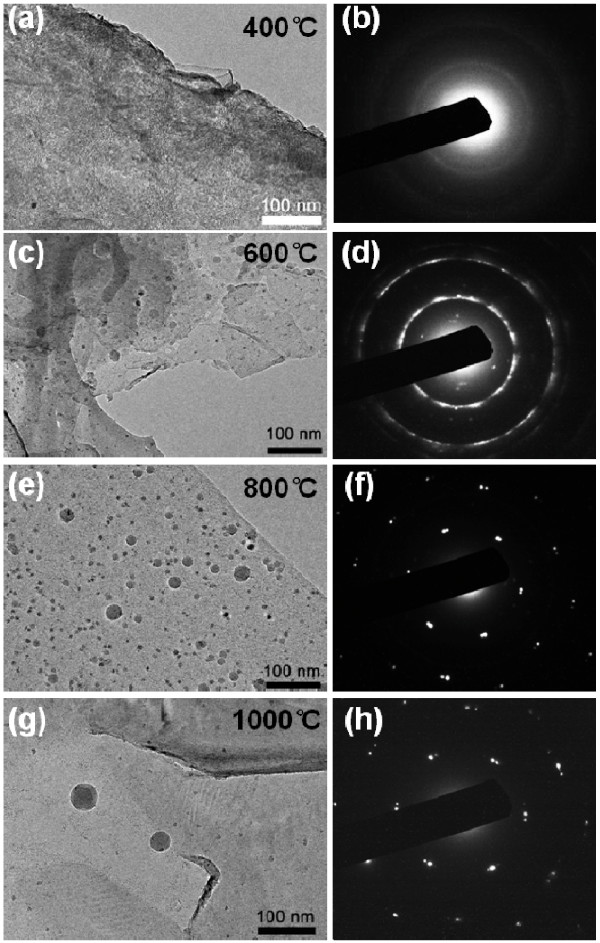
**Typical TEM images and SAED pattern of films obtained at different temperatures.** (**a**, **b**) 400°C, (**c**, **d**) 600°C, (**e**, **f**) 800°C, (**g**, **h**) 1,000°C.

Typical TEM images of films produced at different temperatures are shown in Figure 2. Continuous films were obtained in all the cases (Figure 2a,c,e,g). In addition, there are many ball-like features on the surface of the films, which is similar with a previous report about MLG synthesis on Ni [27]. Considering that the films mainly consist of carbon (XPS spectrum in Additional file 1: Figure S1), these balls are islands of a-C. Moreover, there is an obvious difference in the selected area electron diffraction (SAED) patterns of samples produced at different temperatures, as shown in Figure 2b,d,f,h. The SAED pattern of the 800°C and 1,000°C samples consists of diffraction spots attributable to graphene [28]. The SAED pattern contains multiple spots, which could be caused by back-folding of edges, intrinsic rotational stacking faults, or overlapping domains of graphene layers [28]. Both spots and rings are found in the SAED pattern of the 600°C sample. Since the films mainly consist of carbon, the diffraction spots can be attributed to MLG, and the rings, to a-C. This indicates that a hybrid structure of MLG and a-C is obtained at 600°C, which is also confirmed by scanning electron microscope (SEM) images and Raman spectra (Additional file 1: Figures S2 and S3). Only diffraction rings are found in the SAED pattern of the 400°C sample, indicating that the 400°C sample is an a-C film. 

Different synthesizing temperatures resulted in different films. The reason could be explained as follows: The CVD growth of graphene on a transition metal consists of three stages: (1) precursor molecules collide with the metal surface; (2) carbon precursor molecules dehydrogenate and form active carbon species; and (3) active carbon species coalesce, nucleate, and grow to graphene [16]. In stage 1, the temperatures do not have a significant effect on the reaction because the adsorption energies of organic precursors on the metal surface are very small (approximately 0.02 eV) [16]. At stage 2, the activation energy of liquid organic precursor dehydrogenation is approximately 1.5 eV [16]; thus, a high temperature favors the formation of active carbon species. In stage 3, the high temperature is also beneficial for the graphene growth. At a low temperature, active carbon species lack the mobility to form crystalline carbon, and a more disordered form of carbon is formed [24].

**Figure 3:**
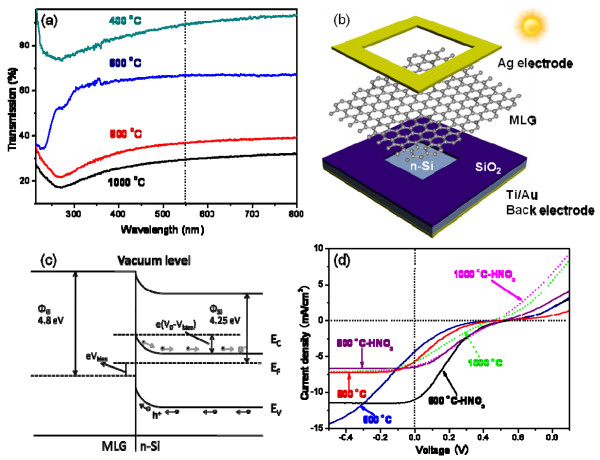
**Optical transmission spectra, solar cell device configuration, energy band diagram, and light current density-voltage curves.** (**a**) Optical transmission spectra of samples produced at different temperatures. (**b**) Schematic diagram of the solar cell device configuration. (**c**) The energy band diagram of the forward-biased MLG/n-Si junction upon illumination. *Φ*_G_ and *Φ*_Si_ is the work function of MLG and n-Si, respectively. *V*_0_ is the built-in potential, and *V*_bias_ is the applied voltage. The photogenerated holes (h^+^) and electrons (e^−^) are driven by *V*_0_ into the MLG and n-Si, respectively. (**d**) Light current density-voltage curves of the solar cells based on 600°C, 800°C, and 1,000°C samples, and the corresponding cells after HNO_3_ treatment.

The optical transmission spectra of the samples synthesized at different temperatures are shown in Figure 3a. Low-temperature samples possess better light transmittance than high-temperature ones. This can be attributed to their thickness difference. High temperature results in high C solubility in Ni [29]; thus, thicker films are supposed to be obtained at higher temperature. The thicknesses of the 600°C and 800°C samples are 25.4 ± 4.1 and 43.7 ± 2.0 nm, respectively (see AFM results in Additional file 1: Figure S4). The *R*_s_ values of samples obtained at different temperatures are measured using a four-probe resistivity test system. The *R*_s_ values of the 400°C, 600°C, 800°C, and 1,000°C samples are 9,564, 1,864, 346, and 262 Ω/sq, respectively. The difference in *R*_s_ between the films prepared at different temperatures is explained as follows:. Firstly, a thicker film forms at a higher temperature. This can be attributed to a higher C solubility in Ni at the higher temperature [29]. The *R*_s_ of thin-film materials is inversely proportional to their thickness [23]. Secondly, it can be seen from the SAED patterns of the four samples (Figure 2b,d,f,g) that high-temperature samples have a higher crystallization degree. This finding that a high temperature improves the crystallization is consistent with that of other reports of graphene synthesis on Ni [19]. A better crystallization can lead to a lower *R*_s_. 

Schottky-junction solar cells are assembled using the 400°C, 600°C, 800°C, and 1,000°C samples and n-type Si. The detailed procedure of the cell assembly can be found in our previous work [23], and a schematic illustration of the solar cell configuration is shown in Figure 3b. The working mechanism of the Schottky-junction solar cells is illustrated using the 800°C sample (MLG). The energy band diagram of the forward-biased MLG/n-Si junction upon illumination is shown in Figure 3c. In this device configuration, MLG serves not only as a transparent electrode for light, but also as an active layer for electron–hole generation, separation, and hole transport. A space-charge region accompanied by *V*_0_ (built-in potential) is formed at the interface of MLG and n-Si because of their work function difference. The photogenerated holes (h^+^) and electrons (e^−^) are driven by *V*_0_ into the MLG and n-Si, respectively. The efficiency of the solar cell based on the 400°C sample is very low, resulting from its poor electrical conductivity. The current density (*J*) versus voltage (*V*) curves of the solar cell based on the 400°C sample is shown in Additional file 1: Figure S5. The *J* versus *V* curves of solar cells based on the 600°C, 800°C, and 1,000°C samples are shown in Figure 3d. The corresponding photovoltaic parameters, such as short-circuit current density (*J*_sc_), open-circuit voltage (*V*_oc_), filling factor (FF), and power conversion efficiency (*η*), are listed in Table 1. As shown in Table 1, solar cells based on the 400°C, 600°C, 800°C, and 1,000°C samples demonstrate power conversion efficiencies of 0.003%, 0.256%, 0.391%, and 0.586%, respectively. 

**Table 1:** Photovoltaic properties of C/Si heterojunction solar cells

**Samples**	***J***_**sc**_	***V***_**oc**_	**FF**	***η***
	**(mA/cm**^**2**^**)**	**(mV)**	**(%)**	**(%)**
400	0.036	419.6	22.7	0.003
400-HNO_3_	0.039	464.9	24.6	0.004
600	4.166	472.4	13.0	0.256
600-HNO_3_	10.932	532.7	18.5	1.080
800	5.504	487.5	14.6	0.391
800-HNO_3_	6.446	517.6	23.97	0.800
1,000	5.67	442.3	23.4	0.586
1,000-HNO_3_	6.33	464.9	27.9	0.820

The power conversion efficiencies of the above-mentioned solar cells are very low, and our previous report had shown that a HNO_3_ treatment could enhance the efficiencies of CNT/n-Si solar cells [30]. To the best of our knowledge, HNO_3_ treatment on MLG/a-C hybrid films had not been investigated yet. We believed that a HNO_3_ treatment may have a similar effect on the above-mentioned solar cells. After the HNO_3_ treatment, the efficiencies of the corresponding solar cells have improved to 0.004%, 1.080%, 0.800%, and 0.820%, respectively, as shown in Table 1. There are three main reasons for the efficiency improvement. Firstly, HNO_3_ doping could enlarge the work function of MLG [31]; thus, a higher *V*_oc_ is obtained after HNO_3_ treatment (Table 1). Secondly, HNO_3_ modification enhances the sheet conductance of the films, leading to a larger *J*_sc_. The *R*_s_ of the 400°C sample decreases from 9,564 to 8,572 Ω/sq, that of the 600°C sample decreases from 1,864 to 1,032 Ω/sq, that of the 800°C sample decreases from 346 to 282 Ω/sq, and that of the 1,000°C sample decreases from 262 to 208 Ω/sq. Thirdly, a HNO_3_ treatment could reduce the internal resistance of the solar cells [30]; thus, the FF is enhanced (Table 1).

The pristine cell efficiencies of the 800°C and 1,000°C samples are better than that of the 600°C sample, and it is interesting that after HNO_3_ treatment, the efficiency of the 600°C sample is better than that of the 800°C and 1,000°C samples. The reasons could be explained by the working mechanism of our Schottky-junction solar cells as follows: Firstly, 600°C, 800°C, and 1,000°C films serve as a transparent electrode for light, and the 600°C sample possesses better light transmittance. Compared with the 800°C and 1,000°C samples, more light reaches to the Schottky-junction interface of the 600°C sample, generating much more electron–hole pairs in the 600°C sample. Secondly, 600°C, 800°C, and 1,000°C films also serve as charge transport path. The electrical conductivity of the 600°C film is much poorer than that of 800°C and 1,000°C ones; thus, the electron–hole pairs could not be effectively separated and transported, resulting in a lower *J*_sc_ and power conversion efficiency. Thirdly, after HNO_3_ treatment, the electrical conductivities of 600°C, 800°C, and 1,000°C films are all improved, enhancing charge transport and *J*_sc_. The 600°C sample generates much more electron–hole pairs, so the magnitude of the increase in *J*_sc_ is much larger than that associated with the 800°C and 1,000°C samples (Table 1), resulting in a better efficiency.

## Conclusions

In summary, the temperature effect on the synthesis of graphitic thin film on Ni foil was investigated. It was observed that temperature was critical in the production of MLG and α-C films. At 400°C, a-C film was obtained on Ni foil by CVD. Hybrid film of MLG and a-C could be synthesized at 600°C, while MLG was obtained at 800°C and above. Schottky heterojunction solar cells based on 400°C, 600°C, 800°C, and 1,000°C samples and n-type Si demonstrated power conversion efficiencies of 0.003%, 0.256%, 0.391%, and 0.586%, respectively. HNO_3_ modification has improved the efficiencies by enhancing the sheet conductance and work functions of the as-synthesized samples. After the HNO_3_ treatment, the efficiencies of 400°C, 600°C, 800°, and 1,000°C devices could be increased to 0.004%, 1.080%, 0.080%, and 0.820%, respectively. Now, low-cost carbon-based window-layer materials have been synthesized and their efficiency can certainly be further improved. Also, carbon-based solar cells are easy to assemble. As such, the commercial production of this type of solar cells holds promise for the future.

## Competing interests

The authors declare that they have no competing interests.

## Authors’ contributions

TC carried out most of the experiments and drafted the manuscript. RL participated in the manuscript preparation. ZH participated in the measurement of the optical transmission spectra. HZ participated in the revision of the manuscript. YJ and SC participated in the solar cell test. FK designed the experiments and revised the manuscript. KW and DW discussed and analyzed the experimental results. All authors read and approved the final manuscript.

## Supplementary Material

Additional file 1 ** Figure S1.** XPS spectrum of 600°C sample. **Figure S2.** SEM images of samples obtained at different temperatures: (a) 600°C, (b) 800°C. **Figure S3.** Raman spectra of 600°C sample. **Figure S4.** AFM images and corresponding height profiles of samples synthesized at different temperatures: (a) 600°C, (b) 800°C. **Figure S5.** The current density (*J*) versus voltage (*V*) curves of solar cell based on 400°C sample and the corresponding cells after HNO_3_ treatment. (DOC 1633 kb) Click here for file
