# Supplementary material for: Low-temperature synthesis of multilayer graphene/amorphous carbon hybrid films and their potential application in solar cells
Source: Nanoscale Res Lett. 2012 Aug 11;7(1):453. doi: 10.1186/1556-276X-7-453 (PMC3479036; doi:10.1186/1556-276X-7-453)
Supplement: Additional file 1 — Figure S1. XPS spectrum of 600°C sample. Figure S2. SEM images of samples obtained at different temperatures: (a) 600°C, (b) 800°C. Figure S3. Raman spectra of 600°C sample. Figure S4. AFM images and corresponding height profiles of samples synthesized at different temperatures: (a) 600°C, (b) 800°C. Figure S5. The current density (J) versus voltage (V) curves of solar cell based on 400°C sample and the corresponding cells after HNO3 treatment. (DOC 1633 kb) [file 1556-276X-7-453-S1.doc]

**Supplementary Information for:**

Low temperature synthesis of multilayer graphene/amorphous carbon hybrid films and their potential application in solar cells

Tongxiang Cui[[1]](#footnote-2), Ruitao Lv^2*^, ZhengHong Huang^1^, Hongwei Zhu^3,4^, Yi Jia^5^, Shuxiao Chen^1^, Kunlin Wang^3^, Dehai Wu^3^, Feiyu Kang^1,5*^

*^1^Laboratory of Advanced Materials, Department of Materials Science and Engineering, Tsinghua University, Beijing 100084, China*

*^2^Department of Physics, The Pennsylvania State University, University Park, PA 16802, USA.*

*^3^Department of Mechanical Engineering and Key Laboratory for Advanced Manufacturing by Materials Processing Technology of Ministry of Education, Tsinghua University, Beijing 100084, China*

*^4^Center for Nano and Micro Mechanics, Tsinghua University, Beijing 100084, China*

*^5^Graduate School at Shenzhen, Tsinghua University, Shenzhen, Guangdong Province, 518055, China*

1. **X-ray photoelectron spectroscopy (XPS) spectra of 600 °C sample**

The X-ray photoelectron spectroscopy (XPS) spectra were collected by a PHI Quantera in a vacuum chamber of 1.4×10^-8^ Torr, using Al K_α_ (1486.7 eV) laser excitation. The spectrum collected from on the 600 °C sample suggests that the sample mainly consists in addition of a small amount of O, as shown in Fig. S1. All Si signals and part of the O signals in the spectrum come from the substrate (Si/SiO_2_); prolonged exposure of the sample to air may have also contributed to the O signal.

**Fig. S1.** XPS spectrum of 600 °C sample.

1. **SEM images of 600 °C and 800** **°C samples**

The surface morphologies of samples are characterized by scanning electron microscopy (SEM, JSM-6460 LV). As shown in Fig. S2, there is an obvious difference between 600 °C and 800 °C samples. The 600 °C sample features overlapping dark and light grey patches (Fig. S2a), suggesting the hybrid nature of as-synthesized films. On the other hand, the surface of the 800 °C sample is homogeneous except for a few border lines.

**
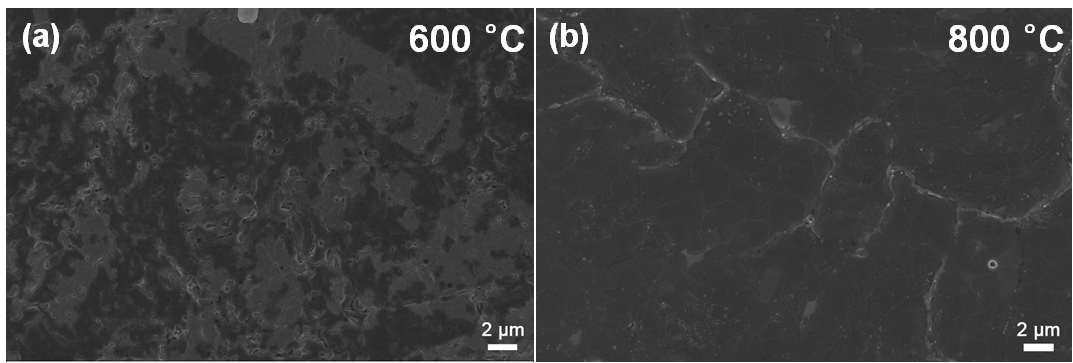
**

**Fig. S2.** SEM images of samples obtained at different temperatures: (a) 600 °C, (b) 800 °C.

1. **Raman spectra of 600 °C sample**

The Raman spectra collected from different areas in 600 °C sample are shown in Fig. S2. Raman spectrum of area 1 shows the feature of multilayer graphene (MLG), while area 2 shows the feature of amorphous carbon.


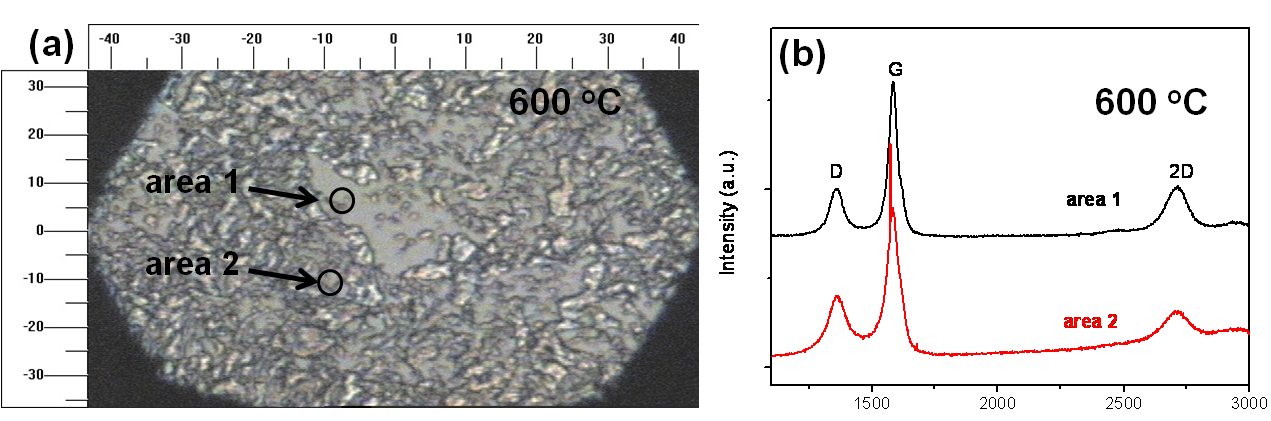


**Fig. S3.** Raman spectra of 600 °C sample.

4. **AFM characterization of 600 and 800 °C samples**

The thicknesses of 600 and 800 °C samples are determined by atomic force microscope (AFM, SPM-9600). AFM images and corresponding height profiles of 600 and 800 °C samples are shown in Fig. S3. The thicknesses of 600 and 800 °C samples are 25.4 ± 4.1 nm and 43.7 ± 2.0 nm, respectively.


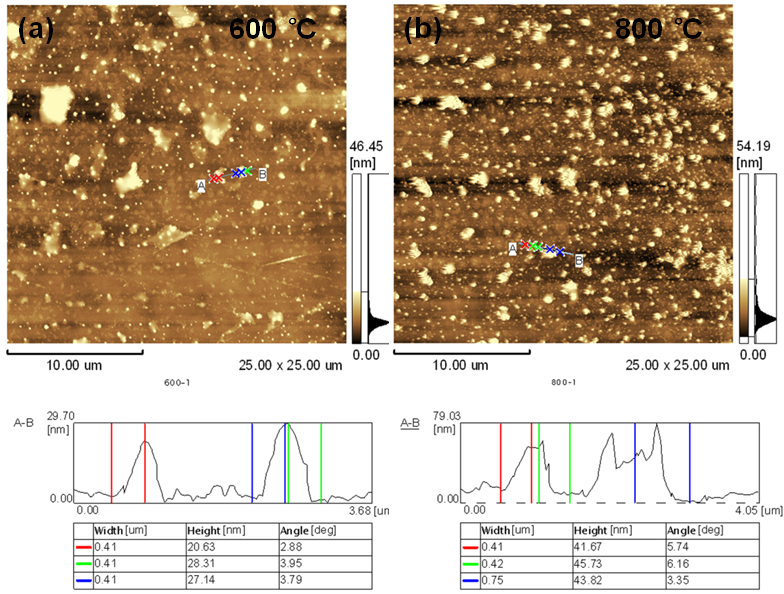


**Fig. S4.** AFM images and corresponding height profiles of samples synthesized at different temperatures. (a) 600 °C, (b) 800 °C. The substrate for AFM measurement is silicon wafer.

**5. The current density (J) versus voltage (V) curves of solar cell based on 400 °C sample**

**Fig. S5.** The current density (*J*) versus voltage (*V*) curves of solar cell based on 400 °C sample, and the corresponding cells after HNO_3_ treatment.

1.  Corresponding authors: fykang@tsinghua.edu.cn (F. Kang) and rxl40@psu.edu (R. Lv). [↑](#footnote-ref-2)
